# Supplementary figures and images for: Notch-induced endoplasmic reticulum-associated degradation governs mouse thymocyte β−selection
Source: eLife. 2021 Jul 9;10:e69975. doi: 10.7554/eLife.69975 (PMC8315795; doi:10.7554/eLife.69975)

Unprocessed images for Figure2-figure supplement 1B

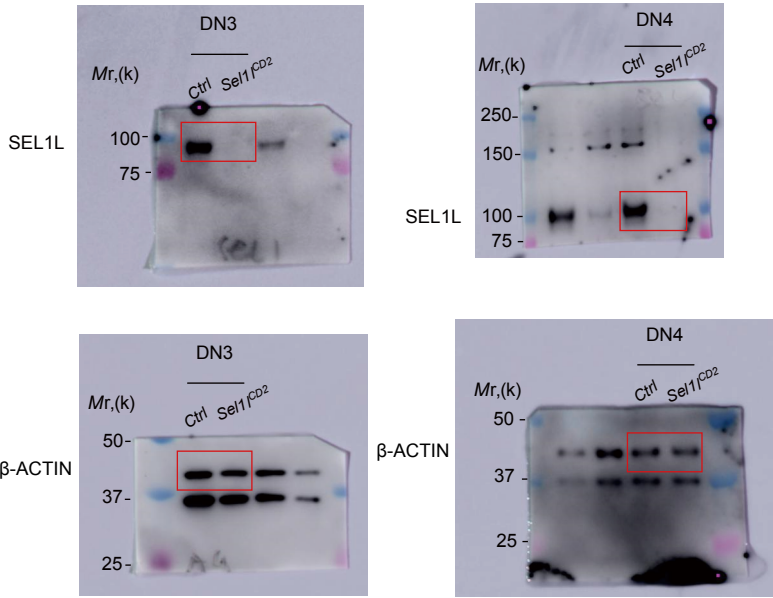

Supplement: Figure 2—figure supplement 1—source data 1. [file elife-69975-fig2-figsupp1-data1.pdf]

Unprocessed images for Figure 4C

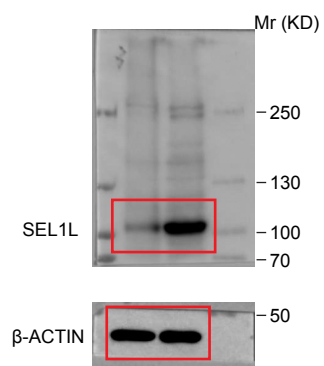

Supplement: Figure 4—source data 1. [file elife-69975-fig4-data1.pdf]

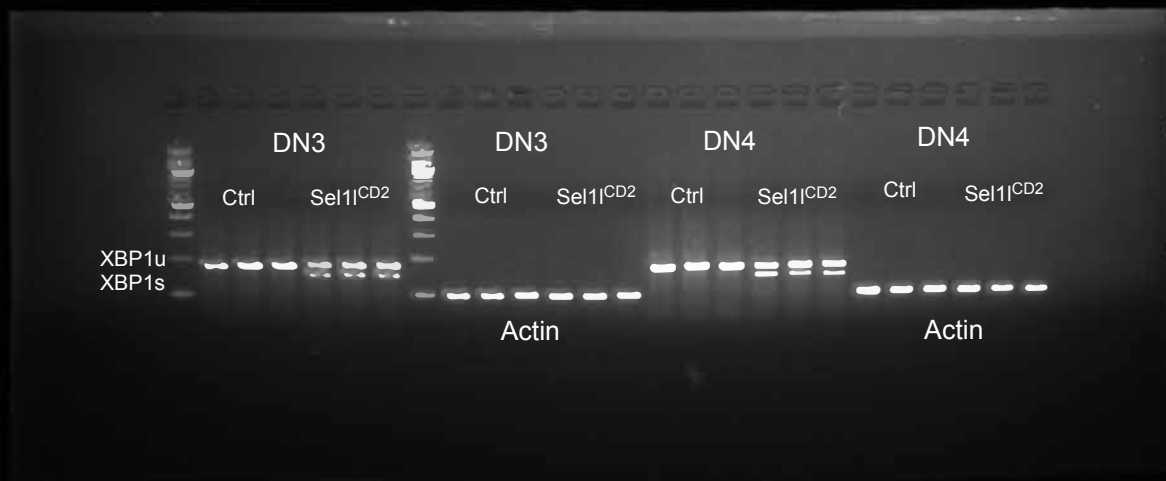

Supplement: Figure 5—source data 2. [file elife-69975-fig5-data2.pdf]

Unprocessed images for Figure5-figure supplement 1A

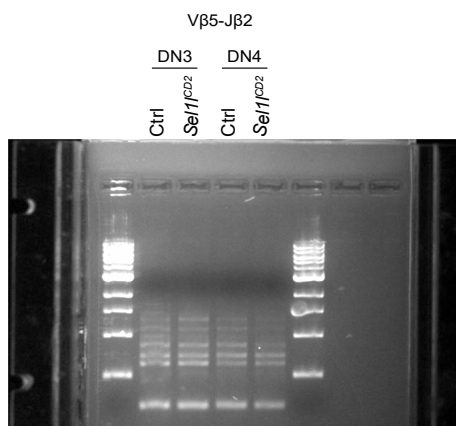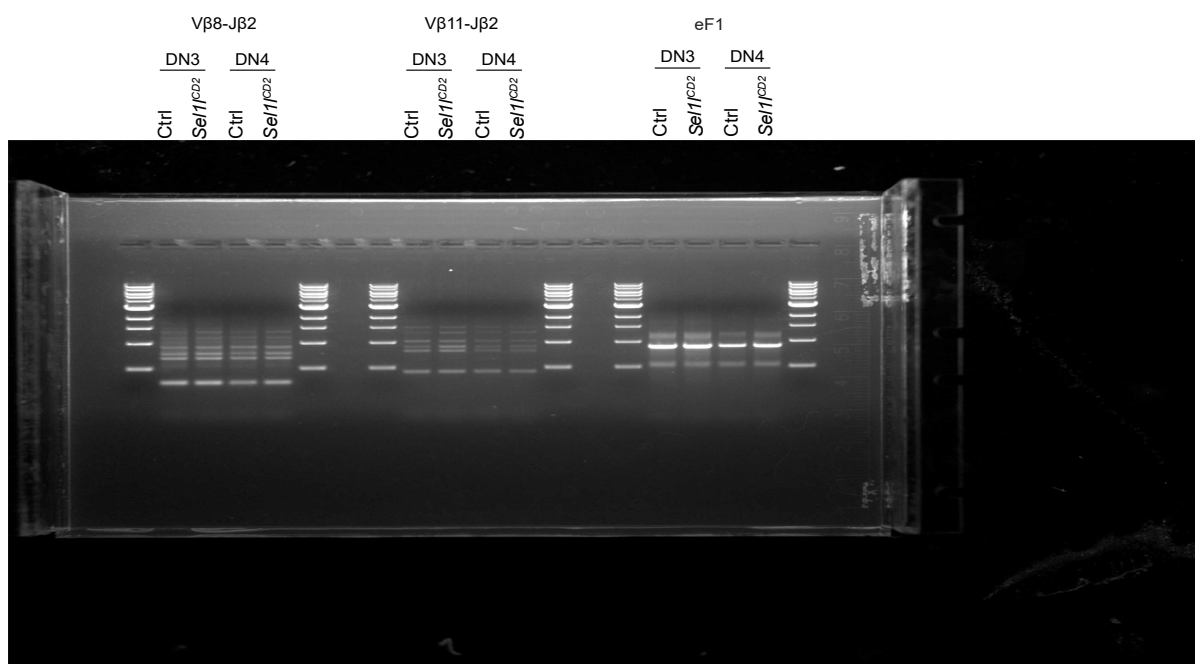

Supplement: Figure 5—figure supplement 1—source data 1. [file elife-69975-fig5-figsupp1-data1.pdf]
