# Supplementary material for: Notch-induced endoplasmic reticulum-associated degradation governs mouse thymocyte β−selection
Source: eLife. 2021 Jul 9;10:e69975. doi: 10.7554/eLife.69975 (PMC8315795; doi:10.7554/eLife.69975)
Supplement: Figure 5—source data 1. [file elife-69975-fig5-data1.pdf]

Unprocessed images for Figure5H

H

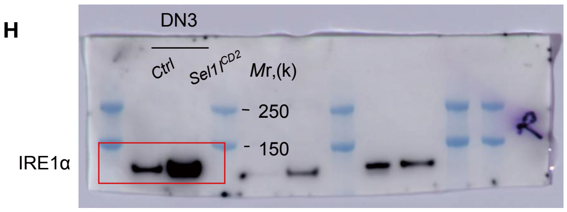

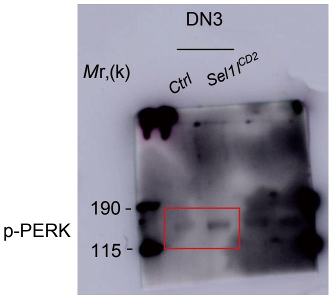

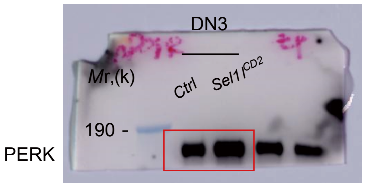

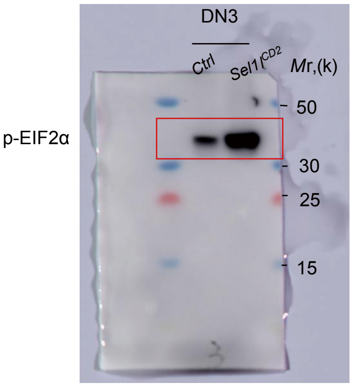

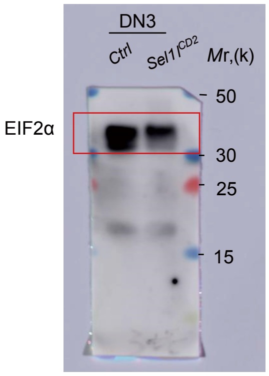

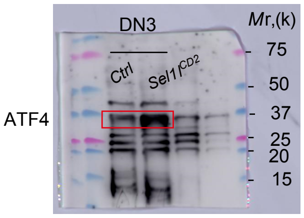

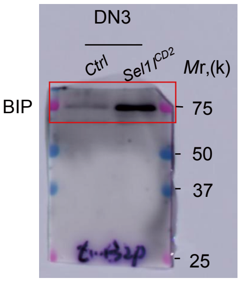

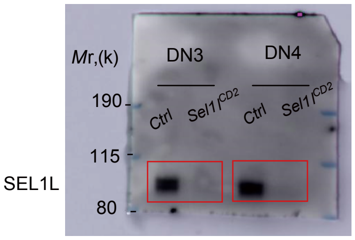

The same western blot images of SEL1L are used in Figure5H, Figure5-figure supplement 1D and Figure5-figure supplement 2A as control because these samples are from the same cell lysates probing with different antibodies.

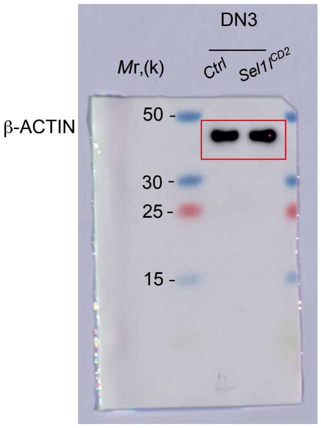

The same western blot images of Actin are used in Figure5H, Figure5-figure supplement 1D as control because these samples are from the same cell lysates probing with different antibodies.
