# Supplementary material for: Notch-induced endoplasmic reticulum-associated degradation governs mouse thymocyte β−selection
Source: eLife. 2021 Jul 9;10:e69975. doi: 10.7554/eLife.69975 (PMC8315795; doi:10.7554/eLife.69975)
Supplement: Figure 5—figure supplement 1—source data 2. [file elife-69975-fig5-figsupp1-data2.pdf]

Unprocessed images for Figure5-figure supplement 1D

Phospho-LCK
(Tyr505)

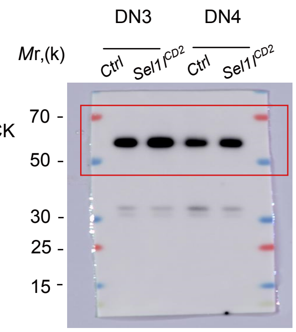

pZAP70
(Tyr319)

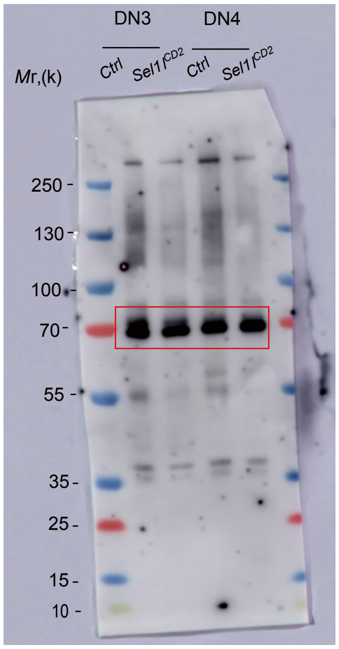

total-ZAP70

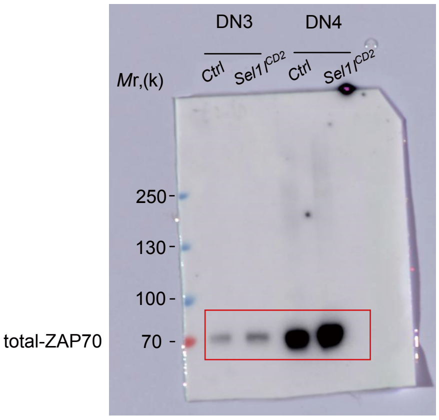

SEL1L

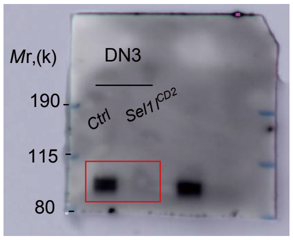

The same western blot images of SEL1L are used in Figure5H, Figure5-figure supplement 1D and Figure5-figure supplement 2A as control because these samples are from the same cell lysates probing with different antibodies.

SEL1L

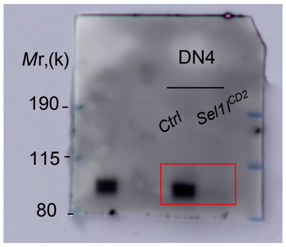

The same western blot images of SEL1L are used in Figure5H, Figure5-figure supplement 1D and Figure5-figure supplement 2A as control because these samples are from the same cell lysates probing with different antibodies.

β-ACTIN

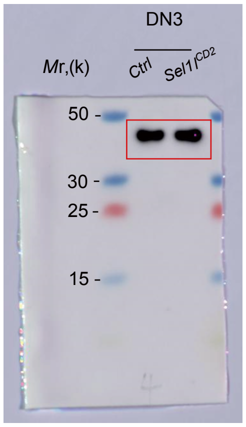

The same western blot images of Actin are used in Figure5H, Figure5-figure supplement 1D as control because these samples are from the same cell lysates probing with different antibodies.

β-ACTIN

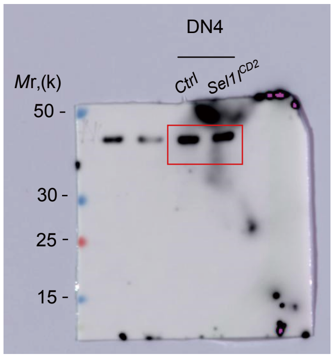

The same western blot images of ACTIN are used in both Figure5-figure supplement 1D and Figure5-figure supplement 2A as control because these samples are from the same cell lysates probing with different antibodies.
