# Supplementary material for: Notch-induced endoplasmic reticulum-associated degradation governs mouse thymocyte β−selection
Source: eLife. 2021 Jul 9;10:e69975. doi: 10.7554/eLife.69975 (PMC8315795; doi:10.7554/eLife.69975)
Supplement: Figure 5—figure supplement 2—source data 1. [file elife-69975-fig5-figsupp2-data1.pdf]

Unprocessed images for Figure5-figure supplement 2A

IRE1a

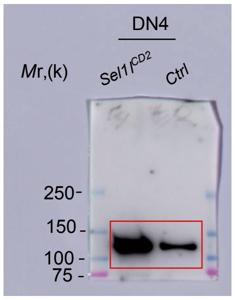

p-PERK

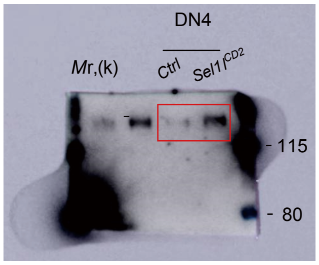

PERK

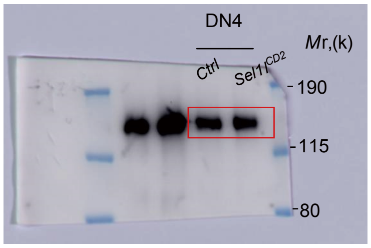

p-EIF2α

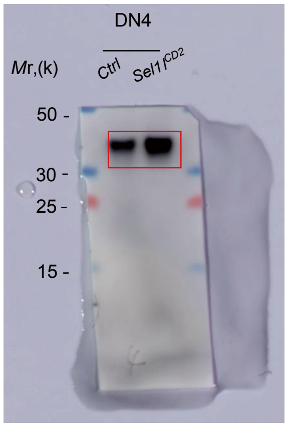

EIF2α

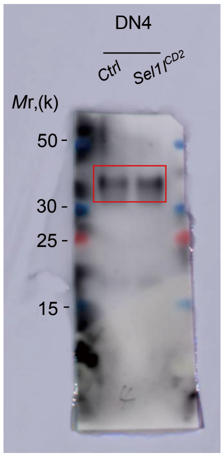

BIP

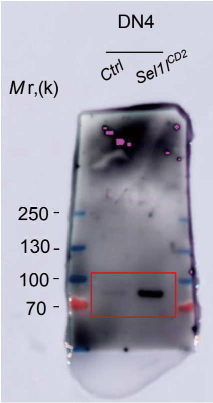

SEL1L

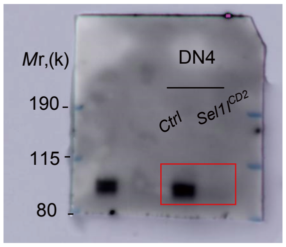

The same western blot images of SEL1L are used in Figure5H, Figure5-figure supplement 1D and Figure5-figure supplement 2A as control because these samples are from the same cell lysates probing with different antibodies.

β-ACTIN

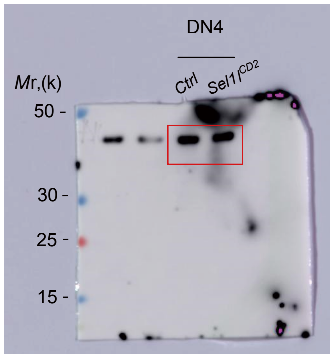

The same western blot images of ACTIN are used in both Figure5-figure supplement 1D and Figure5-figure supplement 2A as control because these samples are from the same cell lysates probing with different antibodies.
